# A retrospective cohort study on the association between early coagulation disorder and short-term all-cause mortality of critically ill patients with congestive heart failure

**DOI:** 10.3389/fcvm.2022.999391

**Published:** 2022-09-16

**Authors:** Yiyang Tang, Qin Chen, Benhui Liang, Baohua Peng, Meijuan Wang, Jing Sun, Zhenghui Liu, Lihuang Zha, Zaixin Yu

**Affiliations:** ^1^Department of Cardiology, Xiangya Hospital, Central South University, Changsha, China; ^2^Department of Neurology, Xiangya Hospital, Central South University, Changsha, China; ^3^National Clinical Research Center for Geriatric Disorders (Xiang Ya), Changsha, China

**Keywords:** heart failure, coagulation disorder, mortality, biomarker, MIMIC-III

## Abstract

**Purpose:**

Coagulation disorder in congestive heart failure (CHF) has been well-documented. The prognostic value of a composite coagulation disorder score, which combines the absolute platelet count, international normalized ratio (INR), and activated partial thromboplastin time (APTT), has not been assessed in CHF. The present study endeavored to explore the association between the coagulation disorder score and adverse outcomes of critically ill patients with CHF.

**Methods:**

Patients diagnosed with CHF in the Medical Information Mart for Intensive Care III (MIMIC-III) database were included in the present retrospective cohort study. The coagulation disorder score was calculated according to the abnormalities of the absolute platelet count, INR, and APTT within 24 h after intensive care unit admission. The primary outcomes were the short-term all-cause mortality, including 30-, 90-day and in-hospital mortalities. The Kaplan–Meier (K-M) survival curve and the Cox proportional hazard model were performed to assess the correlation between coagulation disorder score and outcome events.

**Results:**

A total of 6,895 patients were enrolled in this study and divided into four groups according to the coagulation disorder score. K-M survival curve preliminarily indicated that subjects with higher coagulation disorder score presented lower survival rate and shorter survival time. After adjustment for potential confounders, the multivariate Cox analysis further illustrated that elevated coagulation disorder score as a quartile variable was significantly associated with increased all-cause mortality (quartile 4 vs. quartile 1, 30-day: HR [95% CI], 1.98 [1.50, 2.62], 90-day: HR [95% CI], 1.88 [1.49, 2.37], in-hospital: HR [95%CI], 1.93 [1.42, 2.61]).

**Conclusion:**

In critically ill patients with CHF, ones with high coagulation disorder score tend to be worse clinical prognosis, which would be a promising biomarker and helpful for the management of CHF patients.

## Introduction

Congestive heart failure (CHF) is a clinical syndrome caused by elevated intracardiac pressures and/or deficient cardiac output due to the structural and/or functional abnormality of the heart. Typical symptoms and signs include dyspnea, fatigue, elevated jugular venous pressure, pulmonary congestion, and peripheral edema (1). CHF is a serious manifestation or terminal stage of the development of various cardiovascular diseases with intractable clinical treatment and poor prognosis. The 1-year mortality after diagnosis for patients with all-type heart failure is 20% (2), and patients with severe heart failure admitted to an intensive care unit (ICU) tend to have a worse prognosis with a 1-year mortality of 46.5% (3). The main management principles for advanced and end-stage heart failure are to reduce clinical symptoms, delay progression, avoid decompensation, and control comorbidities (4). As a breakthrough, mechanical circulatory support has emerged as an alternative to heart transplantation and has been proven to improve patient survival and the symptoms of advanced heart failure (5). In addition to efforts to develop new treatments, exploring effective prognostic indicators for severe CHF is important to identify high-risk patients to take timely and effective treatment measures or referral to an appropriate center capable of providing advanced therapies (6, 7).

Similar to other cardiovascular diseases, CHF occurrence and development are also a complicated pathophysiological process that involves many factors, among which the clearer ones include sympathetically increased excitability, activation of the renin–angiotensin–aldosterone system (RAAS), inflammation, myocardial fibrosis, and cardiac remodeling (8, 9). Recently, mounting evidence has shown that patients with CHF have substantial coagulation dysfunction, including platelet activation and hypercoagulability. Thromboembolism has been a common complication in heart failure and tends to increase the risk of cardioembolic stroke and sudden death (10). In patients with CHF, the low-flow state causes abnormal blood flow and promotes blood stasis, and persistent chronic inflammation mediates hypercoagulability. Besides, neurohormonal activation can lead to an overproduction of reactive oxygen species and a deficiency of nitric oxide, which in turn leads to endothelial dysfunction manifested by decreased vasodilation, as well as prothrombotic and proinflammatory states (11–13). Excessive activation of the coagulation system may lead to the consumption of coagulation factors and changes in the corresponding coagulation indexes, which may be closely related to the poor prognosis of patients with CHF and are expected to be promising biomarkers (14).

Disseminated intravascular coagulation (DIC) is characterized by systemic coagulation disorder; is related to sepsis, trauma, and cardiogenic shock; and is associated with an increased risk of death among critically ill patients (15). The DIC scoring system is effective in identifying patients with advanced and possibly irreversible coagulation disorders, which is too late from a treatment point of view. Sepsis-induced coagulopathy (SIC) score composed of platelet count and international normalized ratio (INR) has been proposed to identify the early stage of coagulation abnormalities for early intervention (16). The SIC score has similar performance in predicting the mortality of patients compared with DIC score, and patients could benefit from early anticoagulation according to SIC score when they do not meet the criteria for DIC (17). On this basis, Long et al. reported a composite coagulation disorder score with reference to SIC score and coagulopathy to evaluate early coagulation dysfunction, which combines the platelet count, INR and activated partial thromboplastin time (APTT). This score could effectively predict the risk of atrial fibrillation in patients with sepsis, which is associated with the 90-day mortality of patients (18). The major pathways that contribute to DIC include platelet and inflammatory cell activation and endothelial damage, which are the major hallmarks of heart failure (19). Itani et al. demonstrated that the incidence of DIC in patients with acute heart failure diagnosed with the Japanese Association for Acute Medicine DIC scoring criteria is 5%, and that a higher DIC score is independently associated with increased all-cause mortality (20). However, no study has evaluated the relationship between indicators of early coagulation dysfunction and the prognosis of patients with heart failure. In the present study, clinical data and follow-up from public databases were used to analyze the correlation between early coagulation disorder score and clinical outcome events such as short-term mortality to explore the applicability of coagulation disorder score in prognosis estimation and risk stratification to provide reference for the management of critically ill patients with CHF.

## Materials and methods

### Study design

The present study was a single-center, retrospective cohort study to assess the association between coagulation disorder score and the risk of adverse outcomes in critically ill patients with CHF. The coagulation disorder score was defined by platelet count, INR, and APTT with reference to the sepsis-induced coagulopathy (SIC) score and coagulopathy according to previous studies (**Table 2**) (18). The optimal cutoff was determined using X-tile software (Yale University, New Haven, CT) (21, 22). The primary outcome of the study was short-term all-cause mortality, including 30-, 90-day, and in-hospital mortalities, and the secondary outcomes were the length of ICU stays and the major adverse cardiac events (MACEs) defined as the composite endpoint including all-cause death, readmission for acute heart failure, use of mechanical circulatory support, and implementation of heart transplantation (23).

### Data sources

The anonymized clinical data was extracted from the Medical Information Mart for Intensive Care III (MIMIC-III) database (24). Developed and run by Massachusetts Institute of Technology (MIT), this database included detailed health-related information of more than forty thousand patients who were admitted to the critical care units of the Beth Israel Deaconess Medical Center (BIDMC). In the database, all diagnoses were recorded using the ninth revision of the International Classification of Diseases (ICD-9) code, and the relevant death data including in-hospital and 90-day post-discharge deaths were derived from the inpatient system and the social security database. Notably, the establishment and use of this database has been approved by the Institutional Review Boards of MIT and BIDMC, and the personally identifiable information of subjects has been removed, so additional ethical approval was not essential for the present study.

### Cohort

**Figure 1:**
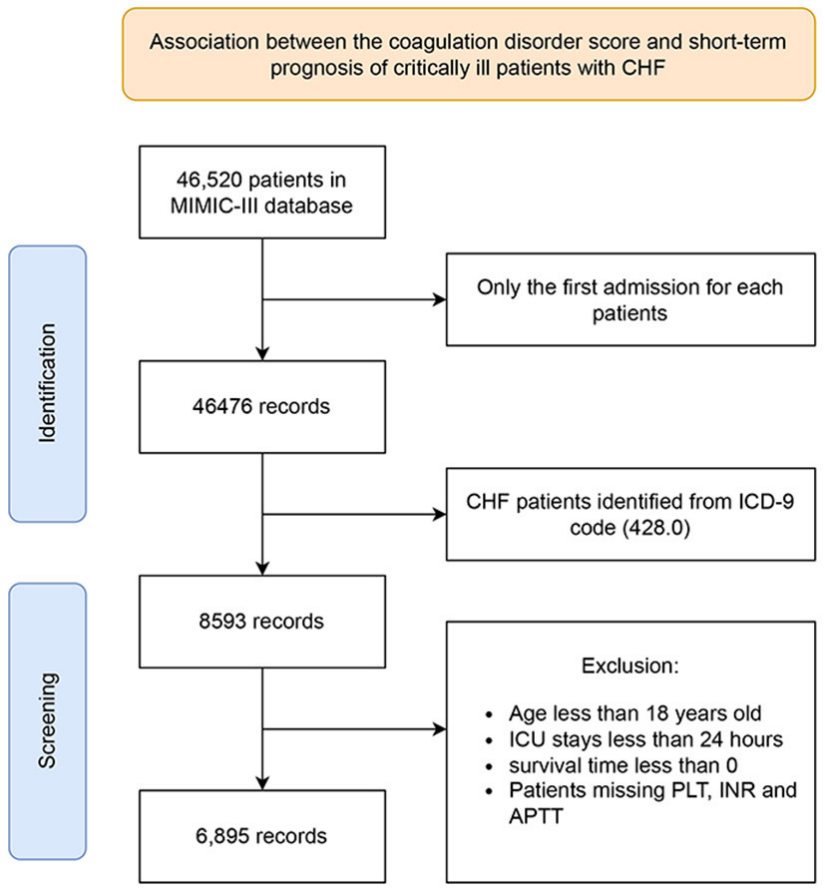
Schematic flow diagram of exclusion and inclusion criteria for selecting subjects. ICU, intensive care unit; CHF, congestive heart failure; PLT, platelet; INR, international normalized ratio; APTT, activated partial thromboplastin time.

Adult patients aged over 18 years old with a diagnosis of CHF (ICD-9 code: 428.0) were selected. Patients not admitted to the ICU and those with an ICU stay of <24 h were excluded. For patients with multiple ICU admissions, only the first was considered. And these patients with missing interest variables (platelet count, INR, APTT) within 24 h after ICU admission were also excluded. Besides, some organ donors may die earlier than the time of admission, resulting in a calculated survival time of <0, and these patients were also excluded. The inclusion and exclusion procedures of the study population were shown in detail in Figure 1.

### Data extraction and preparation

Demographic data, comorbidities, physical finding, laboratory test, severity of illness scores, and therapeutic measures were retrospectively extracted from the MIMIC-III database via structured query language (SQL) with PostgreSQL software (version 9.6, https://www.postgresql.org/). Demographic data consisted of age, gender, and ethnicity. Comorbidities including hypertension, hyperlipemia, diabetes mellitus, atrial fibrillation, acute myocardial infarction (AMI), valvular heart disease (VHD), pulmonary circulation disease (pulmonary embolism and pulmonary hypertension), pneumonia, chronic obstructive pulmonary disease (COPD), liver disease, renal failure, stroke, and malignancy were extracted according to the ICD-9 code recorded in the MIMIC-III database. The sequential organ failure assessment (SOFA) and simplified acute physiology score II (SAPSII) scores were performed to assess the severity. Physical examination included vital signs such as heart rate, respiratory rate, and blood pressure, while laboratory finding included white blood cell count (WBC), hemoglobin, platelet count, glucose, creatinine, sodium, potassium, INR, troponin T (cTnT), N-terminal probrain natriuretic peptide (NT-proBNP), and cardiac index, which was collected within the first 24 h after ICU admission. Therapeutic measures included basic drug therapy for heart failure such as angiotensin-converting enzyme inhibitors (ACEI), angiotensin receptor blockers (ARB), β-blocker, statin, furosemide, and vasopressor, as well as device therapy such as mechanical ventilation, dialysis, and mechanically assisted circulation. Some treatments for coagulopathy were also extracted, including the use of warfarin and heparin, and the transfusion of platelet and fresh frozen plasma (FFP). All data above were extracted by author Tang after completing and passing the CITI “Data or Specimens Only Research” course (No. 9014457).

Regarding the processing of missing values, we adopt two strategies: If the proportion of missing values was <5%, the mean value was used to replace the missing values. When the missing values exceed 5%, the method of multiple compensation was reasonable.

### Data analysis

The continuous variables were reported in the form of mean ± standard deviation (SD) if they conform to a normal distribution, otherwise they were displayed in the form of median [interquartile range (IQR)]. Student's *t*-test (only when normal distribution and homogeneity of variance) or Mann-Whitney U test was conducted to compare continuous variables between survivor and non-survivor groups. Categorical variables were presented as frequencies with percentages and were analyzed with the chi-square test or Fisher's exact test.

Kaplan-Meier (K-M) analysis and log-rank test were used to visualize the differences in 90-day survival of patients with different ranges of platelet, INR, APTT, and coagulation disorder score. Furthermore, Cox proportional hazards models were performed to analyze the association of coagulation disorder score on short-term mortality (i.e., 30-, 90-day, and in-hospital), and results were presented as hazard ratios (HR) and 95% confidence intervals (CI). Logistic regression analysis was used to analyze the association between coagulation disorder score and MACEs, while linear regression models for the correlation of coagulation disorder score with the length of ICU stays. Results were expressed as odds ratio (OR) or β coefficient with the 95% CI for Logistic regression and linear models, respectively. Statistically significant confounders (the effect on interest variables was more than 10%) (25) or clinically important predictors according to past experience were considered as confounders and enrolled in multivariable Cox/logistic/linear regression models, including age, gender, ethnicity, comorbidities (liver disease, atrial fibrillation, and malignancy), vital signs [heart rate, respiratory rate, mean arterial pressure (MBP), urine output, and weight], laboratory tests (WBC, glucose), severity scores (SOFA and SAPSII), treatments (the use of mechanical ventilation, vasopressor, statins, β-blocker, warfarin and heparin, and the transfusion of platelet and FFP).

A stratification analysis was conducted to examine the association of coagulation disorder score and 90-day all-cause mortality between different subgroups stratified by gender, comorbidities (hypertension, hyperlipemia, diabetes mellitus, atrial fibrillation, AMI, VHD, pulmonary circulation disease, pneumonia, COPD, liver diseases, renal failure, stroke, and malignancy), and disease severity scores (SOFA and SAPSII) in the critically ill patients with CHF.

The value of coagulation disorder score, the single indicators, and SOFA score in predicting 90-day all-cause mortality of critically ill patients with CHF was comprehensively assessed by using the area under the receiver operating characteristic, and the sensitivity and specificity.

The statistical analysis above was performed with EmpowerStats software (version 2.20, http://www.empowerstats.com/cn/, X&Y solutions, Inc, Boston, MA) and R software (version 3.4.3). *p* < 0.05 (two-sided) was considered statistically significant.

## Results

### Patient characteristics

A total of 6,895 critically ill patients with CHF were enrolled in the subsequent analysis according to the inclusion and exclusion criteria shown in Figure 1. In the entire study population, the median age was 75.13 years (IQR: 64.49–83.07), 54.37% were male, and 73.52% were Caucasian. After 90 days of follow-up, 1,737 patients died, with a 90-day all-cause mortality rate of 25.19%. The distributions of clinical characteristic variables in the survivor and non-survivor group were shown in Table 1. Compared with the survivor group, subjects in the non-survivor presented more unstable vital signs, including higher heart rate and respiratory rate, and lower blood pressure. Patients in non-survivor appear to be more vulnerable to becoming severely ill, with higher SOFA (median, 4 vs. 6) and SPASII (median, 37 vs. 46) scores. Besides, the clinical situations of these patients tend to be more complicated, and they were more prone to complications such as atrial fibrillation, stroke, renal failure, liver disease, and pneumonia, more likely to use vasoactive drugs, dialysis, mechanical ventilation, etc. to maintain homeostasis. Compared with survivors, patients in the non-survivor had a higher rate of transfusion of FFP, less use of warfarin, and comparable platelet transfusion and heparin use.

**Table 1:** Comparison of baseline data between survivor and non-survivor groups.

**Parameter**	**All**	**Survivors**	**Non-survivors**	***p*-value**
	**(*n* = 6,895)**	**(*n* = 5,158)**	**(*n* = 1,737)**	
**Demographics**				
Age, years	75.13 (64.49–83.07)	73.19 (62.67–81.82)	79.54 (70.30–85.55)	<0.001
Male, *n* (%)	3,749 (54.37%)	2,815 (54.58%)	934 (53.77%)	0.560
Ethnicity, *n* (%)				<0.001
White	5,069 (73.52%)	3,810 (73.87%)	1,259 (72.48%)	
Black	490 (7.11%)	394 (7.64%)	96 (5.53%)	
Others	1,336 (19.38%)	954 (18.50%)	382 (21.99%)	
**Vital signs**				
HR, beats/minute	83.82 (74.08–94.57)	83.42 (74.06–93.71)	85.32 (74.09–97.08)	<0.001
RR, times/minute	18.93 (16.59–21.70)	18.68 (16.44–21.26)	19.90 (17.14–22.97)	<0.001
MBP, mmHg	74.46 (68.76–81.49)	74.92 (69.43–81.72)	73.00 (66.87–80.30)	<0.001
SpO_2_, %	97.37 (95.96–98.52)	97.39 (96.04–98.55)	97.31 (95.67–98.44)	0.001
Weight, kg	77.82 (65.00–92.71)	79.50 (66.55–94.50)	73.00 (61.20–87.20)	<0.001
**Therapies**, ***n*** **(%)**				
ACEI	1,993 (28.91%)	1,666 (32.30%)	327 (18.83%)	<0.001
ARB	293 (4.25%)	252 (4.89%)	41 (2.36%)	<0.001
β-blocker	4,362 (63.26%)	3,433 (66.56%)	929 (53.48%)	<0.001
Digoxin	685 (9.93%)	469 (9.09%)	216 (12.44%)	<0.001
Furosemide	4,820 (69.91%)	3,693 (71.60%)	1,127 (64.88%)	<0.001
Statins	2,467 (35.78%)	2,059 (39.92%)	408 (23.49%)	<0.001
Dialysis	594 (8.61%)	367 (7.12%)	227 (13.07%)	<0.001
Vasopressor	1,729 (25.08%)	1,134 (21.99%)	595 (34.25%)	<0.001
Ventilation	2,281 (33.08%)	1,413 (27.39%)	868 (49.97%)	<0.001
Assisted circulation	418 (6.06%)	299 (5.80%)	119 (6.85%)	0.111
**Laboratory events**				
Hemoglobin, g/dl	11.00 (9.70–12.40)	11.10 (9.70–12.50)	10.70 (9.50–12.00)	<0.001
WBC, K/μl	10.20 (7.40–14.00)	10.10 (7.30–13.60)	10.70 (7.40–15.50)	<0.001
Platelet, K/μl	213.00 (158.00–277.00)	214.00 (161.00–274.00)	211.00 (145.00–283.00)	0.063
Creatinine, mg/dl	1.20 (0.90–1.70)	1.10 (0.90–1.60)	1.30 (0.90–2.10)	<0.001
Glucose, mg/dl	133.00 (115.20–160.84)	131.75 (115.65–157.75)	138.00 (114.00–170.47	<0.001
Sodium, mmol/L	139.00 (136.00–141.00)	139.00 (136.00–141.00)	139.00 (136.00–142.00)	0.029
Potassium, mmol/L	4.20 (3.80–4.60)	4.20 (3.80–4.60)	4.20 (3.80–4.70)	0.015
INR	1.40 (1.20–1.80)	1.40 (1.20–1.70)	1.50 (1.20–2.00)	<0.001
APTT, second	31.70 (27.40–40.70)	31.40 (27.20–40.30)	32.90 (28.00–41.80)	<0.001
NT-proBNP, ng/ml	4.68 (1.78–12.21)	4.40 (1.69–11.49)	5.54 (2.06–14.52)	<0.001
cTnT, ng/mL	0.14 (0.04–0.55)	0.15 (0.04–0.56)	0.13 (0.04–0.49)	0.049
CI, L/min/m^2^	2.15 (1.41–2.99)	2.16 (1.42–3.00)	2.09 (1.35–2.96)	0.041
Urine output, L	1.61 (0.96–2.52)	1.78 (1.11–2.68)	1.15 (0.67–1.91)	<0.001
**Scores**				
SOFA	5.00 (3.00–7.00)	4.00 (2.00–6.00)	6.00 (4.00–8.00)	<0.001
SAPSII	39.00 (31.00–48.00)	37.00 (30.00–45.00)	46.00 (38.00–55.00)	<0.001
**Length of ICU stays, h**	77.00 (46.00–148.00)	73.00 (45.00–132.00)	104.00 (54.00–202.00)	<0.001
**Comorbidities**, ***n*** **(%)**				
Hypertension	1,297 (18.81%)	947 (18.36%)	350 (20.15%)	0.099
Hyperlipemia	2,134 (30.95%)	1,754 (34.01%)	380 (21.88%)	<0.001
Diabetes mellitus	2,459 (35.66%)	1,910 (37.03%)	549 (31.61%)	<0.001
Atrial fibrillation	3,190 (46.27%)	2,285 (44.30%)	905 (52.10%)	<0.001
AMI	563 (8.17%)	425 (8.24%)	138 (7.94%)	0.698
**Comorbidities**, ***n*** **(%)**				
VHD	618 (8.96%)	402 (7.79%)	216 (12.44%)	<0.001
Pulmonary circulation	375 (5.44%)	246 (4.77%)	129 (7.43%)	<0.001
Pneumonia	1,517 (22.00%)	858 (26.60%)	492 (35.65%)	<0.001
COPD	282 (4.09%)	184 (3.57%)	98 (5.64%)	<0.001
Liver diseases	283 (4.10%)	179 (3.47%)	104 (5.99%)	<0.001
Renal failure	1,556 (22.57%)	1,102 (21.36%)	454 (26.14%)	<0.001
Stroke	384 (5.57%)	235 (4.56%)	149 (8.58%)	<0.001
Malignancy	319 (4.63%)	170 (3.30%)	149 (8.58%)	<0.001
**Treatment for coagulopathy**				
Transfusion of FFP	769 (11.2%)	516 (10.00%)	253 (14.57%)	<0.001
Transfusion of platelet	377 (5.5%)	272 (5.27%)	105 (6.04%)	0.2210
Warfarin	430 (6.2%)	374 (7.25%)	56 (3.22%)	<0.001
Heparin	1,577 (22.87%)	1,192 (23.11%)	385 (22.16%)	0.0542

HR, heart rate; RR, respiratory rate; MBP, mean arterial pressure; SpO_2_, percutaneous oxygen saturation; ACEI, angiotensin-converting enzyme inhibitors; ARB, angiotensin receptor blockers; WBC, white blood cell; INR, international normalized ratio; APTT, activated partial thromboplastin time; cTnT, troponin T; NT-proBNP, N-terminal pro brain natriuretic peptide; CI, cardiac index; SOFA, the sequential organ failure assessment; SAPSII, the simplified acute physiology score II; AMI, acute myocardial infarction; VHD, valvular heart disease; COPD, chronic obstructive pulmonary disease; FFP, fresh frozen plasma.

### Primary outcome: Association between coagulation disorder score and mortality

**Figure 2:**
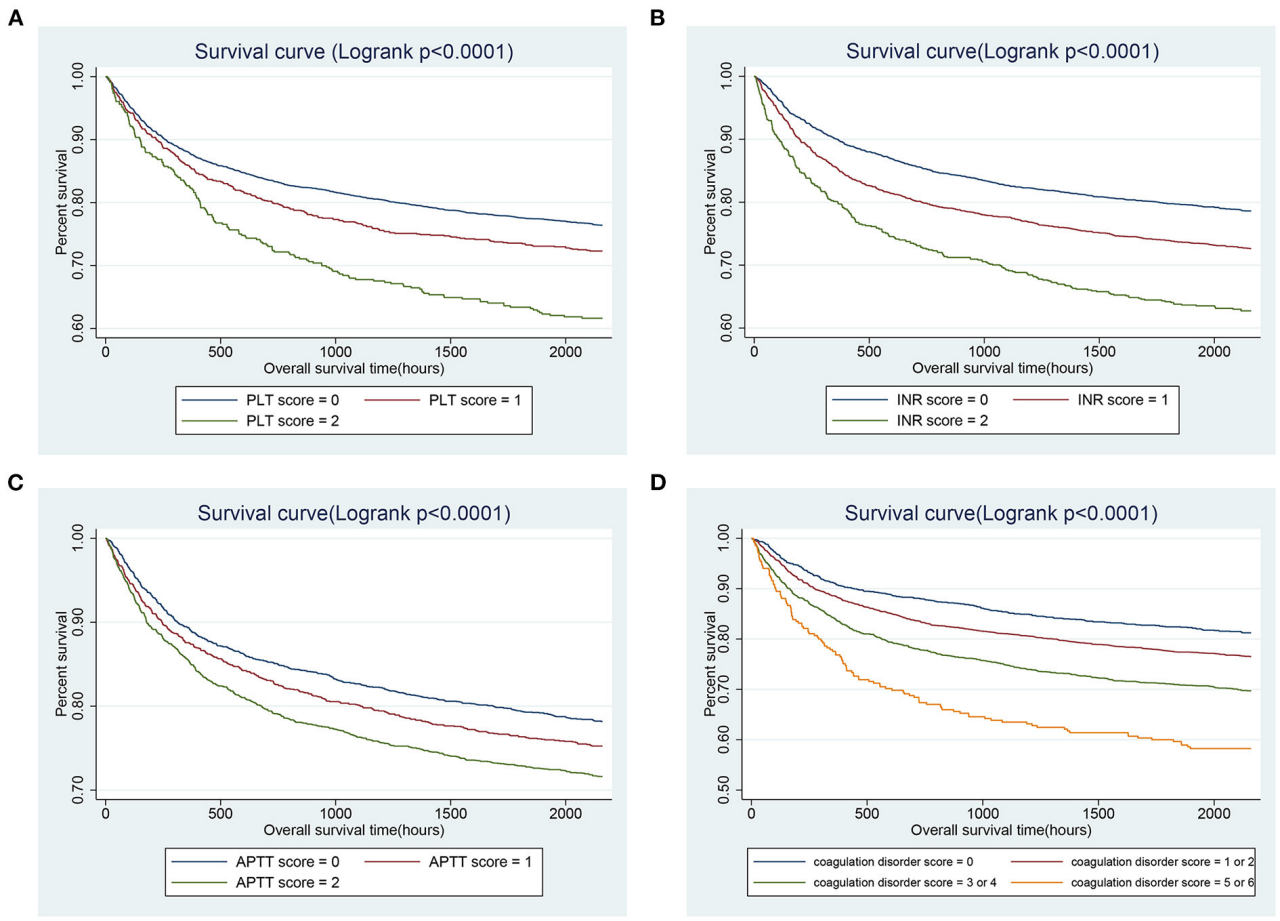
Kaplan–Meier curves of 90-day all-cause mortality among critically ill patients with CHF stratified by PLT **(A)**, INR **(B)**, APTT **(C)**, and coagulation disorder score **(D)**. CHF, congestive heart failure; PLT, platelet; INR, international normalized ratio; APTT, activated partial thromboplastin time.

As shown in Table 2, there were more coagulation abnormalities in the non-survivor group, with lower platelet counts, higher INR and APTT values (*p*-value < 0.05). Hence, the coagulation disorder score was defined by combining platelet, INR and APTT scores. The total coagulation disorder score was 6 points, and the proportions of scores 5 or 6 in the survivor and non-survivor group was 3.22 and 6.85%, respectively, and the difference was statistically significant (*p*-value < 0.05), suggesting that the coagulopathy disorder score may be related to the mortality. The K-M survival curve analysis also showed that patients with a coagulation disorder score of 1 to 6 presented a lower survival rate and a shorter survival time compared to patients with a coagulation disorder score of 0 and the 90-day all-cause mortality increased with the increase in the coagulation disorder score (Logrank test: *p*-value < 0.01, Figure 2). The results of the Cox proportional hazards models of the association between coagulation disorder score and 30-, 90-day, and in-hospital all-cause mortalities were shown in Table 3. In the crude model, patients with a coagulation disorder score of 5 or 6 increased significantly the risk of 30-day (HR, 95% CI: 3.00, 2.33–3.86), 90-day (HR, 95% CI: 2.65, 2.14–3.28), and in-hospital (HR, 95%CI: 2.38, 1.82–3.12) all-cause mortalities with reference to patients with coagulation disorder score of 0. In the model I after adjusting for age, gender, and race, elevated coagulation disorder score was significantly associated with increased 30-day (HR, 95% CI: 3.12, 2.42–4.02), 90-day (HR, 95% CI: 2.71, 2.19–3.36), and in-hospital (HR, 95%CI: 2.79, 2.13–3.66) all-cause mortalities. On the basis of Model I, Model II further adjusted for confounding factors such as comorbidities (liver disease, atrial fibrillation, and malignancy), vital signs (heart rate, respiratory rate, MBP, urine output, and weight), laboratory tests (WBC, glucose), severity scores (SOFA and SAPSII), treatments (the use of mechanical ventilation, vasopressor, statins, β-blocker, warfarin and heparin, and the transfusion of platelet and FFP), and the results showed that coagulation disorder score was still an independent predictor of 30-, 90-day and in-hospital all-cause mortalities, the HRs and 95% CIs were 1.98 (1.50, 2.62), 1.88 (1.49, 2.37), and 1.93 (1.42, 2.61), respectively.

**Table 2:** Comparisons of the abnormalities of PLT, INR, and APTT between survivor and non-survivor groups.

**Parameter**	**All**	**Survivors**	**Non-survivors**	***p*-value**
**PLT score**				<0.001
0 (≥150 K/μl)	5,403 (78.36%)	4,128 (80.03%)	1,275 (73.40%)	
1 (100–150 K/μl)	1,036 (15.03%)	749 (14.52%)	287 (16.52%)	
2 (<100 K/μl)	456 (6.61%)	281 (5.45%)	175 (10.07%)	
**INR score**				<0.001
0 ( ≤ 1.4)	3,759 (54.52%)	2,955 (57.29%)	804 (46.29%)	
1 (1.4–2.6)	2,382 (34.55%)	1,730 (33.54%)	652 (37.54%)	
2 (>2.6)	754 (10.94%)	473 (9.17%)	281 (16.18%)	
**APTT score**				<0.001
0 ( ≤ 29 s)	2,452 (35.56%)	1,917 (37.17%)	535 (30.80%)	
1 (29–34 s)	1,624 (23.55%)	1,222 (23.69%)	402 (23.14%)	
2 (>34 s)	2,819 (40.88%)	2,019 (39.14%)	800 (46.06%)	
**Total score**
**Coagulation disorder type**				<0.001
Coagulation disorder score = 0	1513 (21.94%)	1,229 (23.83%)	284 (16.35%)	
Coagulation disorder score = 1 or 2	3,069 (44.51%)	2,349 (45.54%)	720 (41.45%)	
Coagulation disorder score = 3 or 4	2,028 (29.41%)	1,414 (27.41%)	614 (35.35%)	
Coagulation disorder score = 5 or 6	285 (4.13%)	166 (3.22%)	119 (6.85%)	

PLT, platelet; INR, international normalized ratio; APTT, activated partial thromboplastin time.

**Table 3:** The Cox proportional hazard model of the coagulation disorder score for predicting short-term all-cause mortality in critically ill patients with CHF.

	**30-day all-cause mortality**		**90-day all-cause mortality**		**Hospital all-cause mortality**	
	**HR (95%CI)**	***p*-value**	**HR (95%CI)**	***p*-value**	**HR (95%CI)**	***p*-value**
**Crude**
Coagulation disorder score = 0	1 (ref)		1 (ref)		1 (ref)	
Coagulation disorder score = 1 or 2	1.41 (1.19, 1.67)	<0.001	1.29 (1.13, 1.48)	0.0003	1.23 (1.01, 1.48)	0.0377
Coagulation disorder score = 3 or 4	1.96 (1.65, 2.34)	<0.001	1.76 (1.53, 2.02)	<0.001	1.69 (1.39, 2.05)	<0.001
Coagulation disorder score = 5 or 6	3.00 (2.33, 3.86)	<0.001	2.65 (2.14, 3.28)	<0.001	2.38 (1.82, 3.12)	<0.001
**Model I**
Coagulation disorder score = 0	1 (ref)		1 (ref)		1 (ref)	
Coagulation disorder score = 1 or 2	1.36 (1.15, 1.62)	0.0004	1.25 (1.09, 1.43)	0.0018	1.21 (1.00, 1.47)	0.0487
Coagulation disorder score = 3 or 4	1.91 (1.60, 2.27)	<0.001	1.69 (1.47, 1.95)	<0.001	1.68 (1.39, 2.05)	<0.001
Coagulation disorder score = 5 or 6	3.12 (2.42, 4.02)	<0.001	2.71 (2.19, 3.36)	<0.001	2.79 (2.13, 3.66)	<0.001
**Model II**
Coagulation disorder score = 0	1 (ref)		1 (ref)		1 (ref)	
Coagulation disorder score = 1 or 2	1.38 (1.16, 1.64)	0.0003	1.26 (1.09, 1.45)	0.0013	1.23 (1.01, 1.50)	0.0360
Coagulation disorder score = 3 or 4	1.68 (1.40, 2.01)	<0.001	1.53 (1.32, 1.78)	<0.001	1.51 (1.23, 1.85)	<0.001
Coagulation disorder score = 5 or 6	1.98 (1.50, 2.62)	<0.001	1.88 (1.49, 2.37)	<0.001	1.93 (1.42, 2.61)	<0.001

Crude model was adjusted for none.

Model I was adjusted for age, gender, and ethnicity.

Model II was adjusted for age, gender, ethnicity, liver disease, atrial fibrillation, malignancy, heart rate, respiratory rate, mean arterial pressure, urine output, weight, white blood cell count, glucose, SOFA, SAPSII, the use of mechanical ventilation, vasopressor, statins, β-blocker, warfarin and heparin, and the transfusion of platelet and fresh frozen plasma.

CHF, congestive heart failure; HR, hazard ratio; CI, confidence interval; SOFA, the sequential organ failure assessment; SAPSII, the simplified acute physiology score II.

### Secondary outcome: Association between coagulation disorder score and length of ICU stays and MACEs

The correlation between coagulation disorder score and length of ICU stays and MACEs was also analyzed, and the results were presented in Table 4. In the linear regression models, we observed that patients with a coagulation disorder score of 5 or 6 had a length of ICU stay 33.18 h longer (95%CI: 11.63–54.73) with reference to patients with coagulopathy disorder score of 0, which remained statistically significant (β: 25.83, 95% CI: 4.16, 47.50) after adjustment for potential covariate such as age, sex and comorbidity burden. A similar trend was observed in the multivariate Logistic regression analysis, which indicated that patients with a coagulation disorder score of 5 or 6 had a higher incidence of MAECs (adjusted OR: 2.14, 95% CI: 1.59–2.88).

**Table 4:** The linear and logistic regression analysis of the coagulation disorder score for predicting the length of ICU stays and MACEs in critically ill patients with CHF, respectively.

	**Length of ICU Stays**		**MACEs**	
	**β (95% CI)**	***p*-value**	**OR (95% CI)**	***p*-value**
**Crude**
Coagulation disorder score = 0	0 (ref)		1 (ref)	
Coagulation disorder score = 1 or 2	15.23 (4.75, 25.71)	0.0044	1.48 (1.29, 1.69)	<0.001
Coagulation disorder score = 3 or 4	22.36 (11.03, 33.70)	0.0001	1.97 (1.71, 2.28)	<0.001
Coagulation disorder score = 5 or 6	33.18 (11.63, 54.73)	0.0026	2.58 (1.99, 3.34)	<0.001
**Model I**
Coagulation disorder score = 0	0 (ref)		1 (ref)	
Coagulation disorder score = 1 or 2	15.89 (5.41, 26.37)	0.0030	1.44 (1.25, 1.65)	<0.001
Coagulation disorder score = 3 or 4	23.64 (12.28, 35.00)	<0.001	1.91 (1.65, 2.21)	<0.001
Coagulation disorder score = 5 or 6	31.21 (9.71, 52.70)	0.0044	2.71 (2.08, 3.52)	<0.001
**Model II**
Coagulation disorder score = 0	0 (ref)		1 (ref)	
Coagulation disorder score = 1 or 2	14.75 (4.33, 25.17)	0.0055	1.45 (1.26, 1.69)	<0.001
Coagulation disorder score = 3 or 4	18.33 (6.88, 29.78)	0.0017	1.76 (1.50, 2.07)	<0.001
Coagulation disorder score = 5 or 6	25.83 (4.16, 47.50)	0.0195	2.14 (1.59, 2.88)	<0.001

Crude model was adjusted for none.

Model I was adjusted for age, gender, and ethnicity.

Model II was adjusted for age, gender, ethnicity, liver disease, atrial fibrillation, malignancy, heart rate, respiratory rate, mean arterial pressure, urine output, weight, white blood cell count, glucose, SOFA, SAPSII, the use of mechanical ventilation, vasopressor, statins, β-blocker, warfarin and heparin, and the transfusion of platelet and fresh frozen plasma.

CHF, congestive heart failure; HR, hazard ratio; CI, confidence interval; SOFA, the sequential organ failure assessment; SAPSII, the simplified acute physiology score II.

### Subgroup analysis

Subgroup analysis was further performed to assess the association between coagulation disorder score and 90-day all-cause mortality across different subgroups stratified by gender, comorbidities, and severity of illness scores, as shown in Table 5. The positive correlation between coagulation disorder score and mortality was generally similar across subgroups, with higher scores associated with higher mortality. Remarkably, in the critically ill CHF patients with VHD, stroke, COPD, malignancy, or SOFA <5, patients with a coagulation disorder score of 5 or 6 present the higher risk of death (HR > 1) compared with patients with coagulation disorder score of 0, but which was not statistically significant. No significant interaction was observed in most strata (*p*-value = 0.0712–0.9885), with the exception of gender (*p*-value = 0.0461), and SOFA score (*p*-value = 0.0049). Among critically ill patients with CHF, male patients with a SOFA score of 5 or more had higher risks of 90-day all-cause mortality for high coagulation disorder score.

**Table 5:** Subgroup analysis of the association between the coagulation disorder score and 90-day all-cause mortality.

	**N**	**Coagulation disorder score**				***p* for interaction**
		**0**	**1 or 2**	**3 or 4**	**5 or 6**	
Gender						0.0461
Female	3,146	1.0 (ref)	1.29 (1.07, 1.56)	1.75 (1.44, 2.12)	1.87 (1.31, 2.67)	
Male	3,749	1.0 (ref)	1.33 (1.08, 1.63)	1.83 (1.49, 2.25)	3.37 (2.55, 4.47)	
Hypertension						0.4190
No	5,598	1.0 (ref)	1.35 (1.16, 1.57)	1.76 (1.50, 2.06)	2.73 (2.15, 3.46)	
Yes	1,297	1.0 (ref)	1.08 (0.80, 1.46)	1.73 (1.27, 2.35)	2.33 (1.41, 3.88)	
Diabetes						0.1015
No	4,436	1.0 (ref)	1.27 (1.07, 1.51)	1.83 (1.54, 2.18)	2.35 (1.81, 3.05)	
Yes	2,459	1.0 (ref)	1.31 (1.04, 1.66)	1.57 (1.23, 2.00)	3.36 (2.31, 4.90)	
Hyperlipidemia						0.2395
No	4,761	1.0 (ref)	1.35 (1.16, 1.59)	1.75 (1.49, 2.05)	2.77 (2.19, 3.50)	
Yes	2,134	1.0 (ref)	1.12 (0.84, 1.48)	1.73 (1.29, 2.30)	1.75 (1.00, 3.05)	
AMI						0.2085
No	6,332	1.0 (ref)	1.24 (1.08, 1.43)	1.70 (1.47, 1.97)	2.55 (2.05, 3.17)	
Yes	563	1.0 (ref)	2.20 (1.22, 3.97)	2.85 (1.54, 5.28)	5.62 (2.00, 15.76)	
VHD						0.1233
No	6,277	1.0 (ref)	1.23 (1.07, 1.43)	1.70 (1.46, 1.97)	2.72 (2.18, 3.41)	
Yes	618	1.0 (ref)	1.68 (1.01, 1.97)	2.26 (1.48, 3.45)	1.98 (0.97, 4.07)	
PCD						0.1639
No	6,520	1.0 (ref)	1.24 (1.07, 1.43)	1.72 (1.49, 1.98)	2.54 (2.03, 3.18)	
Yes	375	1.0 (ref)	2.29 (1.34, 3.90)	2.73 (1.54, 4.87)	4.45 (2.08, 9.52)	
Atrial fibrillation						0.0712
No	3,705	1.0 (ref)	1.37 (1.14, 1.65)	1.83 (1.50, 2.24)	2.92 (1.97, 4.17)	
Yes	3,190	1.0 (ref)	1.12 (0.91, 1.37)	1.47 (1.20, 1.80)	1.54 (1.10, 2.16)	
Pneumonia						0.2249
No	5,378	1.0 (ref)	1.25 (1.06, 1.47)	1.82 (1.54, 2.16)	2.75 (2.13, 3.55)	
Yes	1,517	1.0 (ref)	1.38 (1.09, 1.76)	1.61 (1.25, 2.07)	2.39 (1.62, 3.54)	
COPD						0.9885
No	6,613	1.0 (ref)	1.31 (1.13, 1.51)	1.77 (1.53, 2.05)	2.71 (2.18, 3.38)	
Yes	282	1.0 (ref)	1.33 (0.79, 2.22)	1.81 (1.07, 3.06)	2.23 (0.67, 7.41)	
Liver diseases						0.2625
No	4,436	1.0 (ref)	1.28 (1.11, 1.47)	1.75 (1.52, 2.02)	2.30 (1.81, 2.93)	
Yes	283	1.0 (ref)	1.43 (0.55, 3.67)	1.62 (0.64, 4.14)	3.69 (1.44, 9.46)	
Renal failure						0.5722
No	5,339	1.0 (ref)	1.32 (1.13, 1.55)	1.73 (1.47, 2.04)	2.52 (1.96, 3.24)	
Yes	1,556	1.0 (ref)	1.18 (0.89, 1.57)	1.78 (1.34, 2.35)	2.95 (1.96, 4.44)	
Stroke						0.3392
No	6,511	1.0 (ref)	1.36 (1.18, 1.58)	1.86 (1.60, 2.17)	2.91 (2.33, 3.63)	
Yes	384	1.0 (ref)	0.98 (0.66, 1.45)	1.41 (0.93, 2.16)	1.21 (0.29, 5.01)	
Malignancy VHD						0.1971
No	6,576	1.0 (ref)	1.34 (1.16, 1.56)	1.87 (1.61, 2.17)	2.89 (2.31, 3.61)	
Yes	319	1.0 (ref)	1.09 (0.73, 1.62)	1.29 (0.83, 2.00)	1.18 (0.42, 3.31)	
SAPSII						0.1352
<40	3,591	1.0 (ref)	1.28 (1.01, 1.61)	1.36 (1.05, 1.75)	2.16 (1.42, 3.31)	
≥40	3,304	1.0 (ref)	1.24 (1.05, 1.47)	1.74 (1.46, 2.06)	2.40 (1.87, 3.08)	
SOFA						0.0049
<5	3,407	1.0 (ref)	1.17 (0.97, 1.42)	1.25 (1.00, 1.55)	1.02 (0.45, 2.08)	
≥5	3,488	1.0 (ref)	1.29 (1.05, 1.57)	1.81 (1.49, 2.21)	2.43 (1.88, 3.14)	

AMI, acute myocardial infarction; VHD, valvular heart disease; COPD, chronic obstructive pulmonary disease; PCD, pulmonary circulation disease; SOFA, the sequential organ failure assessment; SAPSII, the simplified acute physiology score II.

### Performance of the indicators in predicting 90-day all-cause mortality

This study calculated the area under the curve, sensitivity, and specificity of the coagulation disorder score, platelet, APTT, INR, and SOFA score, as shown in the Table 6. Compared with the single indictors (platelet, INR, and APTT), the coagulation disorder score had the great power for predicting 90-day all-cause mortality of critically ill patients with CHF, as suggested by the largest AUC of 0.609. The sensitivity of coagulation disorder score (45.2%) was higher than platelet count (11.4%) and lower than that of INR (54.2%) and APTT (53.4%). And the specificity of coagulation disorder score (70.4%) was lower than that of platelet count (90.7%) and higher than that of INR (57.2%) and APTT (57.5%).

**Table 6:** Performance evaluation of coagulation disorder score and single indicators in predicting the 90-day all-cause mortality.

**Variables**	**Area under**	**Confidence interval**	**Cut-off**	**Sensitivity**	**Specificity**
	**curve**	**(95%)**			
Platelet	0.538	0.521–0.555	356 K/μl	0.114	0.907
APTT	0.557	0.542–0.573	32.8 s	0.534	0.575
INR	0.572	0.556–0.588	1.4	0.542	0.572
Coagulation disorder score	0.609	0.594–0.624	2	0.452	0.704
SOFA	0.634	0.619–0.649	4	0.647	0.542

INR, international normalized ratio; APTT, activated partial thromboplastin time; SOFA, the sequential organ failure assessment.

## Discussion

As an inevitable end point of almost all cardiovascular diseases, heart failure is dramatically emerging as one of the major public health issues and challenges in the twenty first century with high mortality and rehospitalization rates. Early identification and prognosis evaluation of high-risk patients and intensive treatment are of great importance to improve prognosis (26, 27). Considering the limited ability of symptoms and signs to assess prognosis, the development and application of biomarkers are playing an increasingly important role in the risk stratification of patients with heart failure (28). A series of biomarkers have been proven to be useful for prognosis prediction of heart failure, involving various pathophysiological processes such as inflammation, myocardial injury, remodeling, and oxidative stress. Among them, natriuretic peptides were the most powerful biomarker for the diagnosis and prognosis evaluation of patients with heart failure (29). Besides, soluble suppression of tumorigenicity 2 related to inflammation and fibrosis has become a leading prognostic biomarker for the all-cause and cardiovascular death of acute heart failure, but its application is limited due to the inaccessibility in clinical practice (30, 31). And the effort to identifying some simple and effective indicators is also of significance (32).

In the present study, the clinical data of 6,895 critically ill patients diagnosed with CHF were extracted from the database and were fully analyzed to clarify the importance of the coagulation disorder score in the prognosis assessment of critically ill patients with CHF. The coagulation disorder score is composed of platelet count, INR, and APTT and has the advantages of repeatable and accurate measurement, short test time, and reasonable cost. Our results demonstrated that critically ill CHF patients with high coagulation disorder score tend to have a worse short-term prognosis with considerably increased 30- and 90-day all-cause mortalities. Moreover, positive correlations were observed between coagulation disorder score and the length of ICU stays, as well as the occurrence of MACEs. As a routine test performed in most critically ill patients, the coagulation disorder score is often used to reflect the coagulation status of the body, and the present study further extends the clinical application of this score to provide additional prognostic information for the critically ill patients with CHF.

Patients with heart failure present different degrees of coagulation dysfunction with abnormalities in all components of the Virchow's triad (33). Patients with heart failure have decreased cardiac systolic function and cardiac output, and increased right-sided filling pressures, which aggravate the stasis of blood flow, leading to local ischemia and oxidative stress (34). The disturbance of local blood circulation and neurohumoral activation are involved in the activation of endothelial cells, characterized by a decrease in the release and bio-availability of nitric oxide (NO), which would promote peripheral vasoconstriction and the adhesion of monocytes and platelets to endothelial surfaces (35). Platelet activation induced by high levels of circulating von Willebrand factor in addition to elevated inflammation and oxidative stress contribute to the hypercoagulable state in heart failure (36). Coagulation dysfunction, including DIC and SIC, are common complications in critically ill patients triggered by activation of platelets and inflammatory cells and endothelial damage. As a severe coagulation dysfunction, the incidence of DIC in heart failure is relatively low at about 5%, and the DIC score could independently predict the all-cause death rate (adjusted HR: 2.45; *p*-value = 0.005) (20). SIC is an early coagulation disorder that may deteriorate into DIC, which is often ignored in CHF. In the present study, we found for the first time that the coagulation disorder score with reference to SIC and coagulopathy, composed of platelet count, INR, and APTT value, was positively related to the short-term all-cause mortality of critically ill patients with CHF and has certain clinical application prospect. We should pay more attention to critically ill CHF patients with high coagulation disorder scores, and dynamically assess their coagulation status and be alert to possibility of deterioration into DIC. Moreover, the use of anticoagulant therapy in severe heart failure may need updating. In the present study, patients with high coagulation disorder score were less likely to use anticoagulants such as warfarin (8.53% vs. 2.81%) and heparin (25.38% vs. 14.74%), and had higher rates of transfusions of platelet (2.25% vs. 18.60%) and FFP (1.19% vs. 38.25%), as shown in Supplementary Table 1. These treatment measures did not seem to improve the prognosis of patients, and higher short-term mortality was observed in patients with high coagulation disorder scores (90-day mortality: 18.77% vs. 41.75%), which may be due to the disease severity of these patients or because existing treatment concepts were not suitable. Patients with high coagulation disorder scores may be in an early status of coagulation dysfunction similar to SIC, which may evolve into severe coagulopathy such as DIC without prompt intervention. From the perspective of DIC and SIC, early anticoagulation should be a therapeutic priority in patients with coagulopathy to restore tissue and organ perfusion, and the transfusion of platelets and/or coagulation factors was considered in patients with substantial active bleeding or undergoing invasive procedures and not based solely on abnormal laboratory results (37). The benefit of anticoagulation in patients with heart failure is controversial, especially in the patients without atrial fibrillation (34). Designing and completing clinical trials would be valuable to assess the benefit of early anticoagulation therapy in critically ill CHF patients with early coagulation disorder according to the coagulation disorder score.

The evaluation of predictive performance was also conducted. And the coagulation disorder score composed of platelet, INR, and APTT presented higher predictive power for 90-day all-cause mortality in critically ill patients with CHF, with higher area under the curve compared with single indicators. Besides, the sensitivity and specificity of coagulation disorder score with a cut-off of 2 in predicting 90-day all-cause mortality were 45.2% and 70.4%, respectively, with the middle-ranking sensitivity and specificity compared with single indicators. This result suggested that coagulation disorder score was more likely to identify patients at high risk of death from critically ill patients with CHF compared with platelet count, while the possibility that some low-risk patients may be misjudged as having a high-risk increased. The SOFA score has been widely used as a useful predictor of mortality in the ICU (38), and has been proven to be associated with short-term mortality in acute decompensated heart failure (39). Compared with SOFA score, the coagulation disorder score was slightly inferior for the predictive performance. However, the SOFA score can only be conducted completely after collecting multiple system parameters such as respiration, circulation, and nerves, which limit the convenience of SOFA score compared with coagulation disorder score. The combination of coagulation disorder score and SOFA score or other indicators may well be an option to be considered.

In the subgroup analysis, the positive association of coagulation disorder score with 90-day all-cause mortality did not differ across various subgroups classified by gender, comorbidities, and severity of illness scores, which improved the reliability of the coagulation disorder score in predicting short-term mortality in critically ill patients with CHF. However, the association between coagulation disorder score and mortality in critically ill patients with CHF along with VHD, stroke, COPD, or malignancy had no statistical significance, which may be due to the limited sample size after stratification. Moreover, there was no interaction in most strata, except for gender and SOFA score. Critically ill CHF patients who were male or had a SOFA score of 5 or more with a high coagulation disorder score tend to be at an excess risk of 90-day mortality, which suggested that more attention should be paid to increased coagulation disorder score in these patients. Substantial sex differences in the etiology, clinical manifestations, treatment efficacy, and prognosis of heart failure have been recognized (40). In general, female patients with CHF tend to have a better prognosis compared with men. According to the Framingham Heart Study, the age-adjusted 5-year mortality of women and men with heart failure between 1990 and 1999 were 45 and 59%, respectively (41), which may be related to the cardioprotective effects of estrogen, sex-related differences in the etiology of heart failure, and differences in treatments received and treatment effects (42). No clear evidence supports the remarkable gender differences in coagulation, but Dentali et al. found that men are more likely to bleed during extended treatment of venous thromboembolism with non-vitamin K antagonist oral anticoagulants (43). Besides, as an excellent score for predicting short-term poor-outcome life-threatening conditions, the SOFA score has also been proven to be positively associated with short-term mortality in patients with decompensated heart failure (39).

This study has some limitations. First, this research was a single-center and retrospective cohort study, and the subjects were from the ICU with relatively severe conditions, which limited the representativeness of the study results. Whether the findings of this study can be applied to non-ICU hospitalized patients requires further research, and the conclusions need to be verified in larger prospective cohorts. Second, although some significant variables, including age, comorbidities, and treatments, have been adjusted in the regression model, we cannot provide unavailable confounders in the database, such as specific clinical classifications (heart failure with preserved or reduced ejection fraction), heart function grade in the New York Heart Association Classification and ultrasound cardiac function parameter, which are critical for prognosis. Third, the diagnosis of CHF and other comorbidities was based on the ICD-9 code recorded in the MIMIC-III database, which was relatively sketchy. Fourth, the proportion of DIC in critically ill CHF patients with high coagulation disorder score could not be evaluated, and the comparison of predictive performance for short-term prognosis between coagulation disorder score and DIC score was also not conducted. Lastly, this study only preliminarily analyzed the relationship between the coagulation disorder score and short-term outcome of critically ill CHF patients, did not address the underlying mechanism and intrinsic interaction.

## Conclusion

In the present research, we clarified that the coagulation disorder score was associated with poor outcome in critically ill patients with CHF, including increased short-term all-cause mortality, prolonged ICU stays, and the incidence of MACEs. Besides, the elevated coagulation disorder score occurs in critically ill CHF patients with SOFA score ≥ 5 or men, which requires clinicians to pay more attention.

## Data availability statement

The raw data supporting the conclusions of this article will be made available by the authors, without undue reservation.

## Ethics statement

Ethical review and approval was not required for the study on human participants in accordance with the local legislation and institutional requirements. Written informed consent for participation was not required for this study in accordance with the national legislation and the institutional requirements.

## Author contributions

ZY and LZ conceived and designed the study. YT analyzed the clinical data and drafted the manuscript. QC, BL, and BP assisted with data analysis. MW, JS, and ZL reviewed the study and put forward constructive suggestions. All authors gave final approval of the version to be published and agree to be accountable for all aspects of the work.

## Funding

Our study was supported by the National Natural Science Foundation of China (81873416 and 82100071), the Key Research and Development Program of Hunan Province (2020SK2065), and the Natural Science Foundation of Hunan Province (2022JJ30981).

## Conflict of interest

The authors declare that the research was conducted in the absence of any commercial or financial relationships that could be construed as a potential conflict of interest.

## Publisher's note

All claims expressed in this article are solely those of the authors and do not necessarily represent those of their affiliated organizations, or those of the publisher, the editors and the reviewers. Any product that may be evaluated in this article, or claim that may be made by its manufacturer, is not guaranteed or endorsed by the publisher.
