# Corrigendum: A retrospective cohort study on the association between early coagulation disorder and short-term all-cause mortality of critically ill patients with congestive heart failure

**DOI:** 10.3389/fcvm.2022.1116632

**Published:** 2022-12-15

**Authors:** Yiyang Tang, Qin Chen, Benhui Liang, Baohua Peng, Meijuan Wang, Jing Sun, Zhenghui Liu, Lihuang Zha, Zaixin Yu

**Affiliations:** ^1^Department of Cardiology, Xiangya Hospital, Central South University, Changsha, China; ^2^Department of Neurology, Xiangya Hospital, Central South University, Changsha, China; ^3^National Clinical Research Center for Geriatric Disorders (Xiang Ya), Changsha, China

**Keywords:** heart failure, coagulation disorder, mortality, biomarker, MIMIC-III

In the published article, there was an error in the Funding statement [Our study was supported by the National Natural Science Foundation of China (81873416 and 8210012237), the Key Research and Development Program of Hunan Province (2020SK2065), and the Natural Science Foundation of Hunan Province (2022JJ30981)]. The fund number (8210012237) was the initial acceptance number, not the final grant number. The correct Funding statement appears below.

