# Supplementary material for: A retrospective cohort study on the association between early coagulation disorder and short-term all-cause mortality of critically ill patients with congestive heart failure
Source: Front Cardiovasc Med. 2022 Sep 16;9:999391. doi: 10.3389/fcvm.2022.999391 (PMC9524154; doi:10.3389/fcvm.2022.999391)
Supplement: Supplementary file 1 [file Table_1.DOCX]

**Supplementary Table 1.** The treatment for coagulopathy and mortality in critically ill patients with CHF grouped by coagulation disorder score.

| **Treatment** | **Coagulation disorder score** | | | | ***p* value** |
| --- | --- | --- | --- | --- | --- |
|  | **0** | **1 or 2** | **3 or 4** | **5 or 6** |  |
| Transfusion of FFP | 18 (1.19%) | 243 (7.92%) | 399 (19.67%) | 109 (38.25%) | <0.001 |
| Transfusion of platelet | 34 (2.25%) | 128 (4.17%) | 162 (7.99%) | 53 (18.60%) | <0.001 |
| Warfarin | 129 (8.53%) | 195 (6.35%) | 98 (4.83%) | 8 (2.81%) | <0.001 |
| Heparin | 384 (25.38%) | 749 (24.41%) | 402 (19.82%) | 42 (14.74%) | <0.001 |
| 30-day death | 180 (11.90%) | 503 (16.39%) | 445 (21.94%) | 90 (31.58%) | <0.001 |
| 90-day death | 284 (18.77%) | 720 (23.46%) | 614 (30.28%) | 119 (41.75%) | <0.001 |
| In-hospital death | 142 (9.39%) | 399 (13.00%) | 372 (18.34%) | 85 (29.82%) | <0.001 |

Abbreviations: FFP, fresh frozen plasma; CHF, congestive heart failure.
